# Supplementary material for: ProteinShader: illustrative rendering of macromolecules
Source: BMC Struct Biol. 2009 Mar 30;9:19. doi: 10.1186/1472-6807-9-19 (PMC2672931; doi:10.1186/1472-6807-9-19)
Supplement: Additional file 1 — ProteinShader program without source code. This compressed file contains the complete ProteinShader program including associated libraries, but no source code. A README.txt file gives an overview of the ProteinShader distribution, and the index.html file in the help subdirectory has directions on getting started with the program as well as a set of tutorials. [file 1472-6807-9-19-S1.zip › ProteinShader-beta-0_9_4-binary/help/api/org/proteinshader/structure/io/enums/package-use.html]

Uses of Package org.proteinshader.structure.io.enums (ProteinShader API)


|  |  |  |
| --- | --- | --- |
| **Overview** \| **Package** \| Class \| **Use** \| **Tree** \| **Deprecated** \| **Index** \| **Help** | |  |
| PREV   NEXT | **FRAMES**    **NO FRAMES**     **All Classes** |  |


---


## **Uses of Package org.proteinshader.structure.io.enums**

| Packages that use org.proteinshader.structure.io.enums | |
| --- | --- |
| **org.proteinshader.structure.io.enums** | Holds the enums needed for parsing Protein Data Bank structure files. |

Classes in org.proteinshader.structure.io.enums used by org.proteinshader.structure.io.enums

****CategoryEnum****
  
          This file provides an enumeration of the categories that RecordEnums
fit into.

****RecordEnum****
  
          This file provides an enumeration of the record types defined in the
Protein Data Bank Content Guide (Version 2.2, 20 Dec 1996).

---


|  |  |  |
| --- | --- | --- |
| **Overview** \| **Package** \| Class \| **Use** \| **Tree** \| **Deprecated** \| **Index** \| **Help** | |  |
| PREV   NEXT | **FRAMES**    **NO FRAMES**     **All Classes** |  |


---

# *Copyright © 2007-2008*
